# Prenatal Clinical Assessment of NT-proBNP as a Diagnostic Tool for Preeclampsia, Gestational Hypertension and Gestational Diabetes Mellitus

**DOI:** 10.1371/journal.pone.0162957

**Published:** 2016-09-29

**Authors:** Pawel Sadlecki, Marek Grabiec, Malgorzata Walentowicz-Sadlecka

**Affiliations:** Department of Obstetrics and Gynecology, Collegium Medicum in Bydgoszcz, Nicolaus Copernicus University of Torun, Torun, Poland; Queen's University, CANADA

## Abstract

Common complications of pregnancy include preeclampsia (PE), gestational hypertension (GH) and gestational diabetes mellitus (GDM). Hypertensive disorders (PE/GH) and GDM may result in greater maternal, fetal and neonatal morbidity and mortality. Women with PE/GH, one of the most common causes of heart burden in an obstetrical setting, present with elevated serum levels of BNP and NT-proBNP. The aim of this study was to shed more light on the role of NT-proBNP in pathophysiology of PE, GH and GDM. The study included 156 pregnant women with singleton pregnancies. A total of 26 women developed arterial hypertension during pregnancy, 14 were diagnosed with PE, and GDM was detected in 81 patients. The control group included 35 women with uncomplicated pregnancies, normal arterial blood pressure and normal glucose concentrations. Patients with GH presented with significantly higher serum concentrations of NT-proBNPthan normotensive women (65.5 vs. 37.4 pg/ml; p = 0.0136). Serum levels of NT-proBNP in patients with PE were the highest of all the analyzed subsets, being significantly higher than in women without this condition (89.00 vs. 37.4pg/ml,p = 0,0136). However, women with and without GDM did not differ significantly in terms of their serum NT-proBNPconcentrations. Serum NT-proBNP (pg/ml) (p = 0.0001) and BMI (p<0.0001) turned out to be independent predictors of GH on multivariate logistic regression analysis.Moreover, serum NT-proBNP (pg/ml) was identified as an independent indicator of PE (p = 0.0016). A significant inverse correlation was found between birth weight and maternal serum NT-proBNP concentrations. In our opinion, NT-proBNP can be a useful clinical marker of GH and PE. Determination of NT-proBNP levels may be helpful in identification of patients with PE and GH and in their qualification for intensive treatment; this in turn, may be reflected by better neonatal outcomes.

## Introduction

Common complications of pregnancy include preeclampsia (PE), gestational hypertension (GH) and gestational diabetes mellitus (GDM). Hypertensive disorders (PE/GH) and GDM may result in greater maternal, fetal and neonatal morbidity and mortality [1]. The incidence of these complications has gradually increased worldwide, to 5% for PE, and to 13% for GDM [2,3]. Women who experience these conditions during pregnancy are at increased risk for diabetes mellitus, hypertension and cardiovascular diseases later in life [3]. Recently, pregnant women with PE/GH were shown to present with insulin resistance independent of obesity and glucose intolerance [4]. Previous studies of pregnant women showed that insulin resistance may precede the development of PE, which implies that it is involved in the etiology of the latter [5]. Moreover, a tendency to higher incidence of PE/GH was observed among women with other insulin resistance-related disorders [6]. Several studies showed that women with GDM, as well as patients with polycystic ovary syndrome, are at an increased risk of PE/GH [7]. The risk for PE/GH has been also demonstrated to increase with glucose intolerance [8]. Pathogenic mechanisms of PE/GH include placental hypoperfusion, endothelial impairment, oxidative stress, inflammation and maternal constitutional disorders [9]. Placental vascular dysfunction is also associated with GDM [10,11]. Although all the conditions mentioned above develop before GDM manifests clinically, their sequence and relative contribution to the pathogenesis of the latter are still unknown.

B-type natriuretic peptide (BNP) was first described by Sudoh et al. in1988,after isolation from porcine brain. However, shortly thereafter, it was shown to originate mainly from the cardiac ventricles [12]. Physiologic and pathologic conditions associated with blood volume expansion and/or increased tension on cardiomyocytes lead to upregulation of signals for the synthesis and secretion of cardiac natriuretic prohormones, including A, B, and C type natriuretic peptides [13] Plasma concentrations of cardiac natriuretic peptides, BNP and its N-terminal fragment, proBNP (NT-proBNP), were shown to be sensitive diagnostic markers of mild systolic or diastolic heart failure, asymptomatic left ventricular dysfunction and congestive heart failure in patients with dyspnea treated in an acute care setting [14]. Serum levels of NT-proBNP in pregnancy are higher than in non-pregnant women [15]. Women with PE, one of the most common causes of heart burden in an obstetrical setting, present with elevated serum levels of BNP and NT-proBNP [15,16].

The aim of this study was to shed more light on the role of NT-proBNP in pathophysiology of PE, GH and GDM. We hypothesized that PE, GH and GDM may be associated with some alterations in NT-proBNP levels, and therefore the latter parameter can be used to identify a subset of patients with particularly unfavorable prognosis.

## Materials and Methods

The study was conducted in 2013–2015 at the Department of Obstetrics and Gynecology, LudwikRydygier Collegium Medicum in Bydgoszcz, Nicolaus Copernicus University of Torun. Initially,a total of 188 patients were enrolled, but then women with multiple pregnancies (N = 5), intrauterine growth restriction (N = 3), premature rupture of membranes (N = 5), hypothyroidism (N = 4) and coexisting GH and GDM (N = 10) were excluded, as well as the participants with heart problems and other comorbidities (N = 5). Eventually, the study included 121 patients with singleton pregnancies with complications, among them 26 women who developed GH, 14 diagnosed with PE and GDM was detected in 81 patients. The control group included 35 women with uncomplicated singleton pregnancies, normal arterial blood pressure and normal glucose concentrations.Overall, a total of 156 patients from the study groupand controls were examined.GH was defined as a systolic blood pressure >140 mmHg or diastolic blood pressure >90 mmHg on two or more measurements at least six hours apart, occurring after 20 weeks of gestation, without concomitant proteinuria. PE was defined as the onset of hypertension (systolic blood pressure ≥140mmHg or diastolic blood pressure ≥90 mmHg) in a previously normotensive woman, and proteinuria (at least 0.3 g of protein in a 24-hour urine sample) without a concomitant urinary tract infection. GDM was detected on the basis of a 75 g glucose tolerance test conducted between 24 and 28 weeks of gestation. In all patients, serum NT-pro BNP was determined in a hospital setting, during the third trimester, up to 7 days before delivery. Women presenting with abnormal blood pressure were additionally examined for the severity of hypertension and presence of PE. Patient age, parity, Body Mass Index (BMI), gestational age at delivery, route of delivery,labor induction, birth weight and pH of the umbilical cord blood were analyzed.

10-ml blood samples were obtained from the cubital vein, blood clot was immediately separated by centrifugation and the serum was stored in 1-mL aliquots at -80°C until analysis. Serum NT-proBNP was determined with an electrochemiluminescent sandwich immunoassay with Roche immunoanalyserElecsys® 2010/Cobas e411 (Roche Diagnostics, Manheim, Germany). The between-batch coefficient of variation for the assay is 1.5–4.0% from 148–4423 pg/ml, with analytical range of 5–35000 pg/ml.

Statistical analysis was conducted with PQStat ver. 1.6 software. Non-parametric tests, specifically Kruskal-Wallis test and Mann-Whitney U-test, were used for intergroup comparisons. Relationships between serum concentrations of NT-proBNP (pg/ml),patient age, pH of the umbilical cord blood and birth weight were assessed on the basis of Spearman’s rank correlation coefficient values. Predictors of GH, PE and GDM were identified with univariate and multivariate models of logistic regression.Receiver operator characteristic (ROC) analysis was performed to determinethe discrimination ability of NT-proBNP to recognize GH, PE and GDM.Relationships with p-values < 0.05 and < 0.01 were considered significant and highly significant, respectively.

The protocol of the study was approved by the Local Bioethics Committee at Collegium Medicum in Bydgoszcz, Nicolaus Copernicus University in Torun (decision no. KB 502/2013), and written informed consent was sought from all the study participants.

## Results

Baseline characteristics of the study participants are shown in Table 1.

**Table 1:** Baseline characteristics of the study participants.

Parameter	Study groupN = 121	Control groupN = 35	p
**Age (years)**	29.1 ± 4.6	29.4 ± 5.0	NS
**Parity**	1.9 ± 1.0	1.8 ± 1.0	NS
**BMI (kg/m²)**	25.6 ± 2.0	25.2 ± 2.5	NS
**Pregnancy (weeks)**	38 ± 2.8	40 ± 1.1	NS
**Cesarean sections (%)**	40.6%	37.1%	NS
**pH of umbilical artery**	7.36 ± 0.02	7.35 ± 0.06	NS
**Birth weight (g)**	3298 ± 440	3570 ± 430	NS

(NS–non significant)

No significant intergroup differences were found.

Serum concentrations of NT-proBNP in various subsets of patients are presented in Table 2.

**Table 2:** Serum concentrations of NT-proBNP (pg/ml) in various subsets of patients.

	N	Mean	SD	Min.	Q1	Median	Q3	Max.	Mann-Whitney U-Test
**PE**	14	163.6	173.9	11.3	46.4	89.0	230.4	554.7	Z = 2.47 p = 0.0136
**Controls**	35	45.9	26.6	13.3	26.7	37.4	59.6	109.3	
**GH**	26	134.4	149.5	11.3	32.2	65.5	155.8	499.2	Z = 2.46 p = 0.0136
**Controls**	35	45.9	26.6	13.3	26.7	37.4	59.6	109.3	
**GDM**	81	64.2	69.0	10.5	28.5	51.5	67.6	441.7	NS
**Controls**	35	45.9	26.6	13.3	26.7	37.4	59.6	109.3	

(PE–preeclampsia, GH–gestational hypertension, GDM–gestational diabetes mellitus, Q1 –lower quartile, Q3 –upper quartile, SD—standard deviation, NS–non-significant)

Serum levels of NT-proBNP in patients with PE were the highest of all the analyzed subsets, being significantly higher than in women without this condition (89.00 vs. 37.4pg/ml, Z = 2.47 p = 0.0136). Patients with GH presented with significantly higher serum concentrations of NT-proBNPthan normotensive women (65.5 vs. 37.4pg/ml; Z = 2.46, p = 0.0136). However, women with and without GDM did not differ significantly in terms of their serum NT-proBNP concentrations (p = 0.1818).

ROC analysis was performed to determinethe discrimination ability of NT-proBNP to recognize GH, PE and GDM.The results are summarized in Table 3.

**Table 3:** Relationships between serum concentrations of NT-proBNP and the incidence of gestational hypertension (GH), preeclampsia (PE) and gestational diabetes mellitus (GDM); results of ROC analysis.

Parameter	GH	PE	GDM
**AUC**	0.6481	0.6749	0.4764
**SE(AUC)**	0.0634	0.0923	0.0488
**-95% CI**	0.5239	0.4940	0.3807
**+95% CI**	0.7723	0.8559	0.5720
**Z-statistic**	2.5920	2.1536	-0.4954
**p**	0.0095	0.0313	0.6203
**Cut-off value**	139.7	499.2	21.24

**Fig 1:**
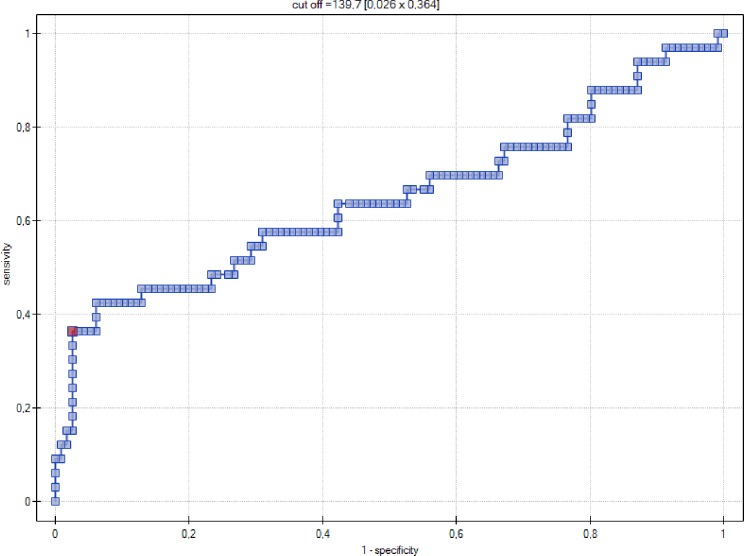
ROC curve–relationship between NT-proBNP levels (pg/ml) and occurrence of gestational hypertension.

Serum concentration of NT-proBNP (pg/ml) turned out to be a significant indicator of GH (p = 0.0095), with the area under ROC curve (AUC) equal to 64% (Fig 1).

**Fig 2:**
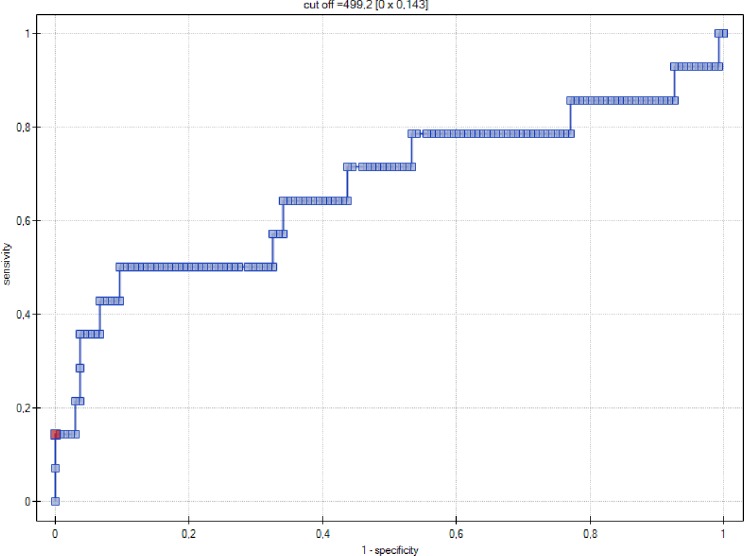
ROC curve–relationship between NT-proBNP levels (pg/ml) and occurrence of preeclampsia.

Moreover, serum level of NT-proBNP(pg/ml) was shown to be a significant indicator of PE (p = 0.0313), with the discriminative value of 67% (Fig 2).

**Fig 3:**
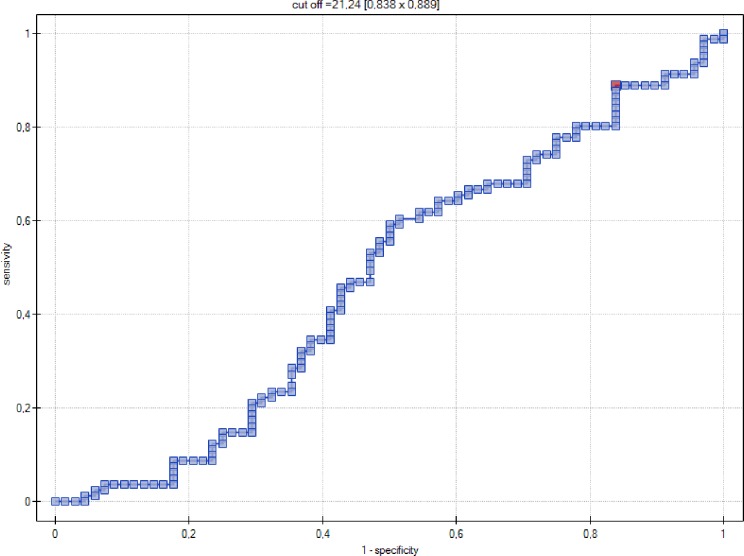
ROC curve–relationship between NT-proBNP levels (pg/ml) and occurrence of gestational diabetes mellitus.

In contrast, serum NT-proBNP (pg/ml) was not a significant indicator of GDM (Fig 3).

Multivariate logistic regression analysis was conducted to verify if the effects of serum NT-proBNP levels on the incidence of GH and PE are independent of other established risk factors of these conditions (Tables 4 and 5).

**Table 4:** Results of multivariate logistic regression analysis examining the effects of serum NT-proBNP levels(pg/ml), BMI, age and primiparity on the incidence of gestational hypertension.

Parameter	b-coefficient	p-value	Odds ratio	-95% CI	+95% CI
**Intercept**	-8.2601	0.0002	0.0003	<0.0001	0.0187
**NT-proBNP (pg/ml)**	0.0094	0.0001	1.0094	1.0045	1.0143
**BMI**	0.1905	<0.0001	1.2099	1.1058	1.3238
**Age (years)**	-0.0039	0.9364	0.9961	0.9058	1.0954
**Primiparity**	0.9267	0.0709	2.5261	0.9242	6.9045

**Table 5:** Results of multivariate logistic regression analysis examining the effects of serum NT-proBNP levels (pg/ml), BMI, age and primiparity on the incidence of preeclampsia.

Parameter	b-coefficient	p-value	Odds ratio	-95% CI	+95% CI
**Intercept**	-2.09	0.3739	0.12	0.001	12.44
**proBNP (pg/ml)**	0.009	0.0016	1.01	1.00	1.01
**BMI**	0.07	0.2265	1.07	0.96	1.20
**Age (years)**	-0.02	0.7519	0.98	0.86	1.12
**Primiparity**	-1.73	0.0160	0.18	0.043	0.72

Serum NT-proBNP (pg/ml) (p = 0.0001) and BMI (p<0.0001) turned out to be independent predictors of GH on multivariate logistic regression analysis.

Moreover, serum NT-proBNP (pg/ml) was identified as a predictor of PE (p = 0.0016) on multivariate logistic regression analysis. Also primiparity turned out to be a significant predictor of preeclampsia in the study group(p = 0.0160).

Relationships between serum concentrations of NT-proBNP and BMI in each study group and in the controls are presented in Table 6.

**Table 6:** Relationships between serum concentrations of NT-proBNP(pg/ml) and BMI in each study group and in the controls.

		N	Mean	SD	Min.	Q1	Median	Q3	Max.	Mann-WhitneyU-Test
**PE**	**BMI <30**	7	55.92	25.72	17.97	36.25	57.78	70.14	106.10	Z = 2.1920p = 0.0284
	**BMI ≥30**	7	22.82	3.84	20.12	20.86	21.32	23.28	28.50	
**GH**	**BMI <30**	10	198.88	176.15	11.32	65.01	155.55	246.57	465.27	Z = 2.3784p = 0.0156
	**BMI ≥30**	16	106.40	130.82	19.47	27.84	54.00	142.44	499.22	
**GDM**	**BMI <30**	38	78.27	90.10	15.34	37.86	57.62	77.43	441.71	NS
	**BMI ≥30**	43	50.36	32.88	10.52	26.69	44.73	64.19	136.55	
**Controls**	**BMI <30**	16	149.95	167.62	11.32	27.25	76.93	225.65	554.70	NS
	**BMI ≥30**	19	118.92	140.23	20.20	30.05	54.65	145.40	499.20	

(PE–preeclampsia, GH–gestational hypertension, GDM–gestational diabetes mellitus, Q1 –lower quartile, Q3 –upper quartile, SD–standard deviation, NS–non-significant)

Patients with BMI ≥30 presented with lower serum concentrations of NT-proBNP than those with BMI <30; however, this relationship was statistically significant solely for patients withPE (21.32 vs. 57.78 pg/ml; Z = 2.1920, p = 0.0284) and GH (54.00 vs.155.55 pg/ml; Z = 2.3784, p = 0.0156), but neither for women GDM nor for the controls.

No significant differences in serum NT-proBNP levels were found between the subsets of patients identified on the basis of their age, parity, gestational age at delivery, route of delivery, labor induction, birth weight and pH of the umbilical cord blood Table 7.

**Table 7:** Serum concentrations of NT-proBNP in the study group, stratified according to selected demographic and obstetrical factors.

NT-proBNP (pg/ml)			N	Mean	SD	Min.	Q1	Median	Q3	Max.	Mann-WhitneyU-Test
**Age(years)**	**PE**	<30	5	37.4	16.4	17.9	23.2	33.5	53.4	61.3	NS
		≥30	9	69.0	35.8	20.1	59.8	75.0	84.2	106.1	
	**GH**	<30	17	42.4	28.0	11.3	23.1	32.6	56.7	499.2	NS
		≥30	9	53.1	27.2	20.1	26.8	35.7	73.5	106.1	
	**GDM**	<30	42	55.4	30.2	12.2	28.2	50.5	77.9	134.7	NS
		≥30	39	63.3	75.2	10.5	22.5	47.7	65.5	441.7	
	**Controls**	<30	20	152.2	174.2	11.3	28.0	62.4	204.9	554.7	NS
		≥30	15	96.9	106.2	20.3	35.0	59.8	101.2	429.3	
**Parity**	**PE**	≥II	5	45.8	32.7	20.1	23.2	34.8	53.4	106.1	NS
		I	9	46.9	23.2	17.9	29.6	46.2	64.2	76.9	
	**GH**	≥II	12	41.9	25.4	20.1	29.0	34.5	49.7	106.1	NS
		I	14	52.8	32.2	11.3	23.9	49.4	68.7	499.2	
	**GDM**	≥II	47	64.4	84.2	11.3	23.0	41.7	63.4	441.7	NS
		I	34	50.0	30.3	10.5	24.5	52.0	64.5	134.7	
	**Controls**	≥II	22	113.0	163.8	13.2	26.8	39.2	82.5	554.7	NS
		I	13	145.5	126.2	11.3	51.0	139.7	204.9	499.2	
**Gestational ageat delivery(weeks)**	**PE**	<38	0	—	—	—	—	—	—	—	—
		≥38	14	46.4	26.5	17.9	23.2	37.8	60.4	106.1	
	**GH**	<38	0	—	—	—	—	—	—	—	—
		≥38	26	46.3	26.6	11.3	26.7	31.2	62.2	499.2	
	**GDM**	<38	10	52.5	13.6	43.8	43.3	51.2	64.9	79.43	NS
		≥38	71	65.7	67.6	10.5	25.1	50.0	65.6	441.7	
	**Controls**	<38	3	152.9	175.0	11.3	28.0	60.2	294.9	499.2	NS
		≥38	32	68.0	103.7	16.0	24.8	59.8	104.5	465.2	
**Route of delivery**	**PE**	Cesarean section	12	75.0	2.7	73.0	74.0	75.0	76.0	76.9	NS
		Vaginal birth	2	41.7	25.6	17.9	21.4	33.5	57.7	106.1	
	**GH**	Cesarean section	14	47.0	21.8	19.7	28.5	42.3	59.3	81.7	NS
		Vaginal birth	12	46.4	33.4	11.3	22.7	31.4	54.9	499.2	
	**GDM**	Cesarean section	38	49.2	27.52	11.3	21.4	42.7	62.7	136.5	NS
		Vaginal birth	43	70.1	81.13	10.5	27.5	50.7	76.4	441.7	
	**Controls**	Cesarean section	12	130.1	144.1	11.3	27.8	65.4	195.5	554.7	NS
		Vaginal birth	23	143.0	193.2	20.3	37.3	47.7	81.2	499.2	
**Labor induction**	**PE**	No	5	45.1	32.1	17.9	21.1	30.4	62.4	106.1	NS
		Yes	9	48.1	19.2	21.1	36.2	49.5	60.4	73.0	
	**GH**	No	11	48.2	30.6	11.3	26.1	39.7	65.4	499.2	NS
		Yes	15	46.1	18.2	21.1	35.4	45.5	59.4	73.0	
	**GDM**	No	21	57.5	30.1	10.5	296.0	54.0	68.9	247.7	NS
		Yes	60	66.5	75.2	11.3	26.9	43.9	70.2	441.7	
	**Controls**	No	14	173.7	177.8	11.3	36.8	101.1	239.3	554.7	NS
		Yes	21	88.5	119.9	16.0	25.9	44.4	74.5	465.2	
**Birth weight(grams)**	**PE**	<2500 g	0	—	—	—	—	—	—	—	NS
		2500–4000	9	46.3	26.5	17.9	26.7	37.8	58.7	106.1	
		>4000	5	47.0	36.7	21.1	34.1	47.0	60.0	73.0	
	**GH**	<2500	2	89.2	62.6	44.7	67.22	85.7	110.2	499.2	NS
		2500–4000	20	48.7	24.4	11.3	26.5	39.2	63.6	109.3	
		>4000	4	33.2	22.6	19.7	23.6	25.2	29.7	73.0	
	**GDM**	<2500	8	69.6	44.4	37.8	43.0	53.0	79.7	134.7	NS
		2500–4000	41	59.4	53.5	10.5	25.9	49.6	62.4	417.0	
		>4000	32	83.1	122.3	15.4	21.3	54.6	71.2	441.7	
	**Controls**	<2500	3	176.8	96.3	11.3	33.6	132.3	446.7	354.7	NS
		2500–4000	28	89.9	109.1	16.0	28.0	54.2	83.9	554.7	
		>4000	4	31.8	6.9	27.8	27.8	47.8	133.8	239.8	
**pH of the umbilical cord blood**	**PE**	<7.26	0	—	—	—	—	—	—	—	—
		≥7.26	14	46.4	26.5	17,9	23.2	37.8	60.4	106,1	
	**GH**	<7.26	0	—	—	—	—	—	—	—	—
		≥7.26	26	50.8	30.6	11,3	29.7	35.8	65.6	499.2	
	**GDM**	<7.26	6	17.3	3.6	19,4	22.9	24.3	25.2	23,1	NS
		≥7.26	75	60.5	69.8	10,5	29.0	53.7	68.5	441,7	
	**Controls**	<7.26	0	—	—	—	—	—	—	—	—
		≥7.26	35	137.6	158.5	11,3	32.2	62.4	195.5	554,7	

(PE–preeclampsia, GH [gestational hypertension, GDM–gestational diabetes mellitus, Q1 –lower quartile, Q3 –upper quartile, SD–standard deviation, NS–non-significant)

**Fig 4:**
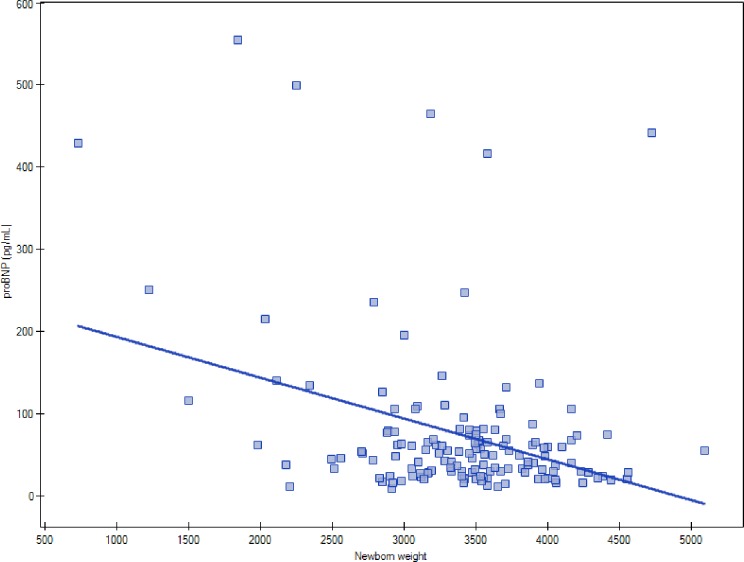
Correlation between NT-proBNP and birth weight.

A significant inverse correlation was found between birth weight and maternal serum concentrations of NT-proBNP(R = -0.2177, p = 0.0088) Fig 4.

Serum levels of NT-proBNP (pg/ml) did not correlate significantly with pregnancy duration or pH of the umbilical cord blood (data not shown).

## Discussion

NT-proBNP is generally used as a marker for diagnosis and monitoring of patients with heart failure [14]. Diagnostic value of NT-proBNP in other conditions associated with cardiac stress, such as pregnancy-associated complications: PE/GH and GDM, is not well established. PE is a complex, hypertensive disorder of pregnancy, demonstrating considerable variability in maternal symptoms and fetal outcomes. Based on the analysis of human placental microarray dataset, Leavey et al. have identified three presumable mechanisms of PE development: “maternal”, with healthy placentas and term deliveries; “canonical”, exhibiting expected clinical, ontological and histopathologic features of PE; and “immunologic” with severe fetal growth restriction and evidence of maternal anti-fetal rejection[17]. It is well known that PE is characterized by a marked increase in peripheral vascular resistance which is reflected by an increase in blood pressure [18]. In uncomplicated pregnancy, peripheral vascular resistance is the highest at early stages and then decreases starting from the 24th week [19]. In turn, serum concentration of NT-proBNP was shown to be elevated in early pregnancy and to return to its pre-pregnancy values before 24 weeks of gestation [20]. Resnik et al. compared serum levels of BNP in women with uncomplicated pregnancies and patients with PE; they suggested that elevated levels of BNP observed in the latter group may reflect left ventricular stress and subclinical cardiac dysfunction related to preeclampsia [21]. Borghiet al. compared serum levels of BNP in pre-eclamptic and healthy pregnant women and demonstrated that elevated levels of this parameter were associated with an increase in left ventricular mass, left ventricular end-systolic and end-diastolic volumes, as well as with a significant reduction of the left ventricular ejection fraction [22]. This implies that the increase in serum NT-proBNP observed during the course of PE may occur secondarily to enhanced cardiac load. Alternatively, increased cardiac load and elevated serum levels of NT-proBNP/BNP may be two independent components of a relatively heterogeneous preeclampsia syndrome. Moreover, it should be remembered that a fraction of circulating NT-proBNP/BNP is released from the placenta [23].Our findings are consistent with the results of previous studies in which patients with PE and GH presented with significantly higher serum levels of NT-proBNP than women with uncomplicated pregnancies. Furthermore, we showed that elevated serum level of NT-proBNP is a significant indicatorof PE and GH. In our opinion, NT-proBNP can be a useful clinical marker of PE and GH. Determination of NT-proBNP levels may be helpful in identification of patients with PE and GH and in their qualification for intensive treatment; this in turn, may be reflected by better neonatal outcomes.

Women with GDM are at increased risk of PE and other pregnancy-related complications. Patients with PE/GH show insulin resistance during pregnancy [24,25]. Furthermore, several studies demonstrated that women with a history of PE/GH may present with enhanced insulin resistance even years after delivery, also after controlling for body mass index and upon exclusion of patients with a history of GDM [26]. Similarly, women with a history of PE/GH may display specific features of metabolic syndrome, a condition associated with insulin resistance, years after delivery [27,28]. Finally, either women with PE or patients with GDM may show endothelial dysfunction and markers of chronic vascular inflammation, both during and after pregnancy [29]. However, we did not find significant differences in serum concentrations of NT-proBNP in patients with GDM and the controls, and this parameter was not identified as aindicator of GDM. Our findings are consistent with the results published by Andreas et al. who also did not find significant differences between patients with GDM and controls in terms of their NT-proBNP levels. Consequently, this biochemical marker of heart failure may not be sensitive enough to detect early stages of impaired cardiac function,when routinely used reference ranges are applied. Therefore, the risk of a false negative result needs to be considered in patients with GDM [30].

Obesity at the time of pregnancy is associated with increased risk of gestational hypertensive disorders and GDM [31].In our present study, patients with PE or GH and BMI ≥30 presented with significantly lower concentrations of NT-proBNPthan women with BMI<30; however, similar differences were not observed in women with GDM and in the controls. Moreover, both serum concentration of NT-proBNPandBMI were identified as significant indicators of gestational hypertension in our study group. Relative abundance of natriuretic peptide clearance receptors (NPR-C) in adipose tissue has been proposed as amechanism contributing to the decreaseinnatriuretic peptide concentration during the course of obesity [32]. On the other hand, plasma levels of NT-proANP and NT-proBNPin obese subjectsare reduced to a comparable degree as their mature peptides. Sincethe pro-peptides were not demonstrated to bind to NPR-C, this is impairment of theirsynthesis or secretion which likely plays a role in obesity[33].

Parity and mode of delivery may also influence plasma levels of NT-proBNP, as circulating volume increases after birth, inducing synthesis of natriuretic peptides [34]. According to Yamada et al., a considerable proportion of puerperal women may exhibit a transient modest increase in NT-proBNP level. This phenomenon was particularly evident in primiparas, especially after caesarean delivery, and was less likely to occur after vaginal delivery in multiparous women. This suggests that cesarean section is associated with additional volumetric overload [34]. Based on the umbilical cord blood analysis, Won Joon et al. concluded that stressful intrauterine environment may stimulate fetuses to synthesize NT-proBNP. According to the same authors, vaginal delivery is not associated with an increase in umbilical cord blood NT-proBNP levels and consequently, does not promote a fetal cardiac stress [35].In our present study, serum concentration of NT-proBNP did not show significant associations with parity and route of delivery. Moreover, NT-proBNP levels were not modulated by participants’ age, gestational age at delivery, birth weight and pH of the umbilical cord blood.

NT-proBNP was previously shown to play a significant role during fetal life, especially with regards to organogenesis of the cardiovascular system, regulation of blood pressure and water balance in the developing embryo, and transition from intra- to extra-uterine life [36]. A growing body of evidence points to NT-proBNP as a biomarker for screening, diagnosis, management and follow-up of children with cardiac disease, bronchopulmonary dysplasia, retinopathy and increased risk of intraventricular hemorrhage (IVH) [37–40]. Multivariate analysis revealed that serum concentration of NT-proBNP in our patients correlated inversely with birth weight of their offspring.However, we did not find significant birth weight-related differences in the concentrations of NT-proBNPin women from any of the study groups.

We are well aware of potential limitations of this study. Relatively small size of the sample does not allow us to establish any ultimate conclusions. Consequently, larger studies are needed to confirm the hereby presented findings and to explore the hereby proposed etiopathogenic mechanisms.
